# CDK7 as a Potential Exploratory Biomarker for Distinguishing Acute Myocardial Infarction Subtypes via DDR Pathways: Evidence From a Bangladeshi Cohort

**DOI:** 10.1002/clc.70383

**Published:** 2026-06-10

**Authors:** Rifat Hossain Ripon, Hasnat Zahin, Abdullah Al Noman, Abdullah Al Saba, Mohammad Sayem, A.H.M. Nurun Nabi, Tahirah Yasmin

**Affiliations:** ^1^ Department of Biochemistry and Molecular Biology, Laboratory of Population Genetics University of Dhaka Dhaka Bangladesh

**Keywords:** AMI, DDR, gene expression, NSTEMI, STEMI

## Abstract

**Introduction:**

Acute myocardial infarction (AMI) is a major cause of cardiovascular death, with STEMI and NSTEMI as distinct subtypes. Current diagnostic tools often lack the sensitivity and timeliness to rapidly distinguish these subtypes at early clinical presentation. The DNA damage response (DDR), activated by ischemia and oxidative stress during AMI, represents a promising pathway for identifying novel biomarkers. Despite evidence of its role in myocardial infarction, little is known about DDR gene expression in South Asian populations, particularly in Bangladesh. This study evaluated the expression of four DDR genes; ATM, CDK7, OGG1, and NBN and their potential for distinguishing between STEMI and NSTEMI.

**Methods:**

A total of 70 AMI patients and 60 healthy controls from the Bangladeshi population were recruited. RNA was extracted from blood samples, and gene expression was quantified using qRT‐PCR. Statistical analyses included Mann–Whitney U tests for group differences, linear regression analysis and Spearman's correlation analysis for gene co‐expression.

**Results:**

CDK7 expression was significantly higher in NSTEMI compared to STEMI patients (*p* < 0.05). However, ATM, OGG1, and NBN did not differ significantly between the subtypes. Co‐expression analysis revealed strong positive correlations among all four genes, with the strongest between ATM and CDK7 (r = 0.76, *p* < 0.001).

**Conclusion:**

This is the first study in the Bangladeshi population to demonstrate distinct DDR gene expression patterns across AMI subtypes, with CDK7 showing subtype‐specific differential expression between STEMI and NSTEMI. Although the observed discrimination was modest and requires further validation, these findings provide important exploratory insights into ischemia‐associated transcriptional regulation in an underrepresented population.

## Graphical abstract

Differential expression of DNA damage response genes was evaluated in Bangladeshi AMI patients. CDK7 showed significantly higher expression in NSTEMI compared to STEMI, while ATM, OGG1, and NBN showed no subtype‐specific differences, highlighting potential transcriptional heterogeneity between AMI subtypes.

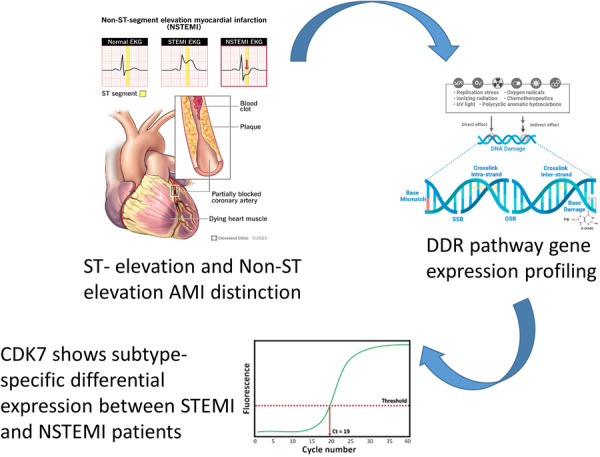

**Abbreviations**
CVD: cardiovascular disease
MI: myocardial infarction
AMI: acute myocardial Infarction
cTn: cardiac troponin
ECG: electrocardiogram
STEMI: ST‐ elevation myocardial infarction
NSTEMI: non‐ST‐elevation myocardial infarction
DDR: DNA damage response
Ct: cycle threshold
PBMCs: peripheral blood mononuclear cells

## 1 Introduction

Myocardial infarction (MI) refers to heart muscle injury caused by insufficient blood supply, usually due to acute ischemia. A more severe and clinically significant form of this condition is Acute Myocardial Infarction (AMI), or heart attack, which occurs when a coronary artery is abruptly blocked, often from thrombus formation or plaque rupture, leading to oxygen deprivation and tissue damage [1]. The diagnosis of AMI primarily relies on a dynamic rise and/or fall in cardiac troponin levels above the 99th percentile, alongside clinical signs including chest pain, ECG changes, or imaging evidence of new injury [1]. In 2021, AMI was responsible for approximately 7 million deaths and 129 million DALYs (Disability‐Adjusted Life Year; a metric used to quantify the burden of disease) worldwide [2]. This represents a substantial global economic burden and highlights the urgent need for early detection and intervention strategies. AMI can be further classified based on ECG into ST‐elevation myocardial infarction (STEMI), characterized by complete artery blockage, significant ECG changes, and elevated biomarkers such as high‐sensitivity troponins (hs‐cTnI or hs‐cTnT) and CK‐MB, and non‐ST‐elevation myocardial infarction (NSTEMI), caused by partial occlusion, often lacking clear ECG changes and diagnosed mainly by rising and falling troponin levels without STEMI [3].

While cardiac troponins are the primary diagnostic indicators for myocardial infarction, they are not used alone for subtype discrimination; rather, the distinction between STEMI and NSTEMI relies heavily on the ECG. However, ECG changes can be subtle or absent in NSTEMI, and troponin elevation often occurs several hours after the initial injury. Consequently, there is a need for molecular biomarkers that can rapidly distinguish these subtypes and reflect early cellular stress. In recent years, the focus of cardiovascular biomarker research has shifted beyond traditional protein‐based markers toward molecular biomarkers, particularly those based on gene expression, because they can detect early cellular stress responses before irreversible damage occurs and offer greater specificity in complex clinical presentations.

One of the key molecular consequences of AMI is the generation of intense oxidative stress, which damages not only lipids and proteins but also DNA within cardiomyocytes and circulating immune cells. This DNA damage activates the DNA damage response (DDR), a highly conserved cellular network that detects, signals, and repairs genetic lesions to maintain genomic stability and prevent cell death [4]. The human genome encodes more than 450 genes involved directly or indirectly in DDR pathways, encompassing damage sensors, signal transducers, DNA repair enzymes, cell cycle regulators, and apoptotic effectors. These genes function through several well‐established repair mechanisms, including base excision repair (BER), nucleotide excision repair (NER), mismatch repair (MMR), homologous recombination (HR), and non‐homologous end joining (NHEJ). DDR gene expression depends on the tissue type and the kind of cellular damage, such as the ischemic injury that occurs during AMI. Consequently, profiling DDR gene expression offers a promising strategy not only for the early molecular detection of myocardial injury but also for gaining insight into how effectively cardiac or immune cells are responding to ischemic stress. Beyond this, DDR gene expression profiles may also serve as molecular tools for classifying AMI subtypes, such as STEMI and NSTEMI. Given that the extent and nature of DNA damage likely differ between these subtypes due to variations in ischemic duration, severity, and reperfusion dynamics, it is likely that distinct DDR activation patterns exist between them. Therefore, exploring the expression of DDR genes in this context could not only improve early detection but also help distinguish between these two subtypes, which is critical for timely therapeutic decision‐making and risk stratification.

Recent transcriptomic studies have identified several DDR‐related genes whose altered expression correlates with ischemic injury and disease progression in AMI patients. A previous study has reported altered expression of DNA damage response genes in coronary artery disease patients, with ATM expression showing significant association with fibrous plaque morphology in NSTEMI patients [5]. Additionally, oxidative stress pathway analyses in STEMI patients identified ATM as a downregulated gene during myocardial ischemia/reperfusion injury, suggesting involvement of DNA damage signaling in acute myocardial infarction [6]. NBN, a key component of the MRE11/RAD50/NBN DNA double‐strand break repair complex, has also been shown to regulate ATM recruitment and DNA repair kinetics following oxidative DNA damage [7]. These observations suggest that up or down regulation of these genes can create a critical bottleneck that could stall the heart's ability to fix genomic damage during an acute event. However, these studies were conducted in a non‐Bangladeshi population, and gene expression is known to be influenced by ethnicity, genetic background, and environmental factors, raising questions about the generalizability of those results to other populations. Given their known functional roles in the DDR network and their prior association with cardiovascular stress, ATM, CDK7, OGG1, and NBN were selected for targeted expression analysis in this study.

Each gene represents a key node in distinct yet interconnected DDR mechanisms. ATM (Ataxia Telangiectasia Mutated) is a master regulator of the cellular response to DNA double‐strand breaks and oxidative stress, modulating repair, apoptosis, and inflammatory signaling [8]. CDK7 (Cyclin‐Dependent Kinase 7) functions at the crossroads of cell cycle control and transcription regulation, supporting myocardial regeneration under stress conditions [9]. OGG1 (8‐Oxoguanine DNA Glycosylase 1) is a key enzyme in the base excision repair (BER) pathway that removes oxidized DNA bases, such as 8‐oxoguanine, which accumulate during ischemia. NBN (Nibrin), part of the MRN complex (MRE11‐RAD50‐NBN), is critical for sensing double‐strand breaks and coordinating homologous recombination repair [10]. Despite the promising findings from earlier research, no study has yet investigated the expression of these DDR genes in different AMI patients' subgroups within the Bangladeshi population. Therefore, this study aims to evaluate and compare the expression levels of ATM, CDK7, OGG1, and NBN between STEMI and NSTEMI subtypes of AMI patients. By doing so, this study explores the potential of DDR genes not only as early, non‐invasive diagnostic biomarkers but also as molecular classifiers that may aid in AMI subtype differentiation and patient risk stratification.

## 2 Methods

### 2.1 Study Design

This study employed a cross‐sectional, case‐control design to investigate differences in ATM, CDK7, NBN, and OGG1 gene expression between STEMI and NSTEMI patients in the Bangladeshi population. Peripheral blood samples were collected, total RNA was extracted, and gene expression was quantified using real‐time quantitative PCR (qPCR). Expression levels were normalized to the housekeeping gene GAPDH to account for variations in RNA quality and quantity, enabling reliable comparison between groups.

### 2.2 Study Participants

The study involved 70 patients diagnosed with acute myocardial infarction (AMI) who were admitted to the Cardiology Department of Dr. Sirajul Islam Medical College Hospital in Dhaka, Bangladesh. The patients were selected based on diagnostic criteria such as elevated troponin levels, angiogram findings, and ECG reports. In addition, 60 healthy control individuals were included in the study who had no previous history of cardiovascular disease or other chronic illnesses. Ethical approval for this study was obtained from the Ethical Approval Committee of the Faculty of Biological Sciences, University of Dhaka. All participants filled out a questionnaire to gather their demographic information and health measurements by consent. Blood samples were collected from the patients immediately, within 4 h of their admission into the hospital under strict aseptic conditions. During the transportation time, a 4°C–8°C temperature was maintained.

### 2.3 Gene Expression Analysis via qRT‐PCR

RNA extraction was carried out within 1 h of blood sample collection to maximize the RNA output. RNA was extracted from whole blood using the RNAsimple Total RNA Kit (TIANGEN BIOTECH (BEIJING) CO., LTD, catalog: 4992858) according to the manufacturer's instructions. The quality and quantity of the extracted RNA were evaluated using the NanoDrop OneC Micro volume UV–Vis Spectrophotometer (Thermo Fisher Scientific, US) and the extracted RNA was stored at −80°C. Afterwards, cDNA was constructed from the extracted RNA using Takara PrimeScript™ 1st strand cDNA Synthesis Kit (catalogue: 6110 A). The cDNA was diluted in nuclease‐free water to minimize inhibition caused by excessive template amounts, which can affect qPCR performance for most genes. qRT‐PCR was used to measure the expression of the DDR genes, along with glyceraldehyde‐3‐phosphate dehydrogenase (GAPDH) serving as the reference gene. The reactions were carried out on a StepOnePlus™ Real‐Time PCR System (Applied Biosystems) using the Takara TB Green Premix Ex Taq (Tli RNaseH Plus) (Cat. No. RR420L) as the master mix. The qPCR primers used are given in Supporting Information S1: Table 1. Primer specificity was confirmed using melting curve analysis.

Relative quantification of mRNA levels of the target genes was analyzed using the 2^−ΔΔCt method. First, the Ct (cycle threshold) values for the target genes and the housekeeping gene GAPDH were obtained from the qRT‐PCR analysis. Then, the ΔCt was calculated for each individual subject (both patients and controls) as:

ΔCt (subject) = Ct (target gene, subject) − Ct (GAPDH, subject)

Next, the mean ΔCt of the healthy control group (*n* = 60) was calculated for each target gene:

Mean ΔCt (control) = [Σ ΔCt (control subjects)]/60

For each subject, the ΔΔCt was then calculated as:

ΔΔCt (subject) = ΔCt (subject) − Mean ΔCt (control)

The relative fold change in gene expression was then computed using the formula:

Relative expression (fold change) = 2 ^–ΔΔCt^.

Finally, log_2_‑transformed fold‑change values were derived as log_2_ (2^−ΔΔCt), and all statistical comparisons between STEMI and NSTEMI were performed on these per‑subject log_2_ fold‑change values.

### 2.4 Statistical Analysis

All the statistical analyses were performed using the R programming language. Numeric variables were represented by Mean (±SD) values. The Shapiro‐Wilk test was used to check whether the data set of this study has a normal distribution. Linear regression was used to adjust per‑subject log_2_ fold‑change values for age, BMI, hypertension, diabetes, smoking, and family history of CVD. For each gene, residuals from the model [log_2_FC ~ confounders] were extracted as confounder‑adjusted expression values. Mann–Whitney *U* tests compared STEMI versus NSTEMI on both unadjusted and adjusted log_2_ fold‑changes. This non‐parametric test was selected for its appropriateness in comparing independent groups without the presumption of a normal distribution.

Model formula: Model_CDK7 <‐ lm(log2_fold_CDK7 ~ Age + BMI + Hypertension + Diabetes + smoking + CVD_history, data = data_patients)

data_patients$adjusted_CDK7 <‐ resid(model_CDK7)

Mann–Whitney *U* tests:

Before adjustment: wilcox.test(log2_fold_CDK7 ~ Group, data = data_case)

After adjustment: wilcox.test(adjusted_CDK7 ~ Group, data = data_case)

Spearman's Rank Correlation was performed to explore the correlation between fold change of all four genes in ST‐elevation myocardial infarction and non–ST‐elevation myocardial infarction patients. To explore the potential clinical discriminatory ability of CDK7, receiver operating characteristic (ROC) curve analysis was performed, and the area under the curve (AUC) with 95% confidence interval was calculated. Logistic regression was used to evaluate the added value of age and sex.

## 3 Result

### 3.1 DDR Gene Expression Comparison Between STEMI and NSTEMI Bangladeshi Patients

#### 3.1.1 *ATM* Expression

**Figure 1:**
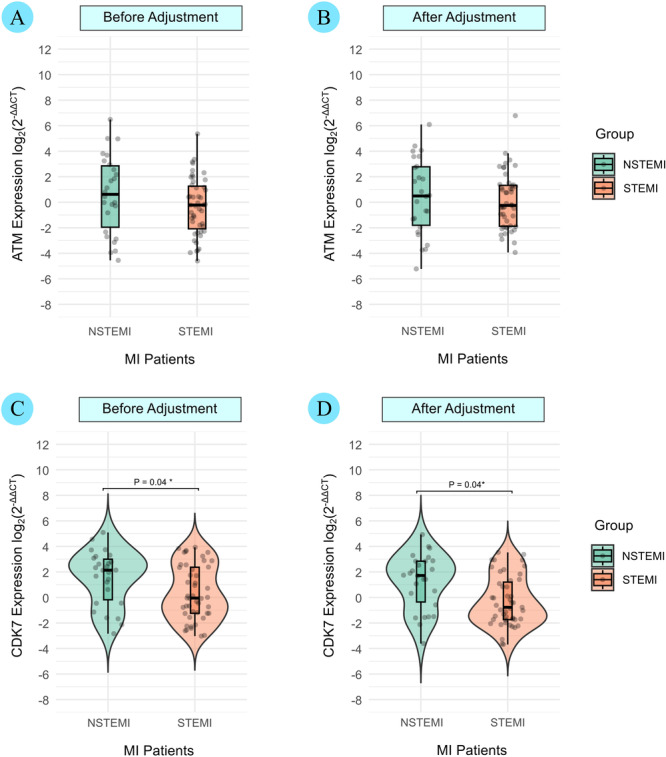
ATM and CDK7 gene expression in STEMI and NSTEMI patients. Box plots showing the relative expression of ATM and CDK7 genes (log2 (2^^−ΔΔCt^)) in STEMI and NSTEMI patients. A) ATM expression before adjustment for potential confounders and (B) after adjustment. (C) CDK7 expression before adjustment and (D) after adjustment (*, **, and *** represent *p* < 0.05, < 0.01, and < 0.001 respectively).

Before adjustment (Figure 1A), the median log‐transformed ATM expression was higher in NSTEMI patients (0.621) compared with STEMI patients (–0.219), although this difference did not reach statistical significance (*p* = 0.16). After adjusting for potential confounders (Figure 1B), the median ATM expression decreased slightly in both groups, with NSTEMI showing a log median of 0.493 and STEMI a log median of –0.462. The *p*‐value increased to 0.42, indicating that the difference between the two groups became even less statistically significant following adjustment.

#### 3.1.2 *CDK7* Expression

Before adjustment (Figure 1C), the median log‐transformed CDK7 expression was significantly higher in NSTEMI patients (2.14) compared with STEMI patients (–0.0473; *p* = 0.04). After adjusting for potential confounders, the difference persisted, with slightly reduced median values in both groups (NSTEMI: 1.82; STEMI: –0.774) and remained statistically significant (*p* = 0.04) (Figure 1D). These findings indicate that NSTEMI patients consistently exhibit higher CDK7 expression than STEMI patients, even after accounting for potential confounding variables. This persistent difference may reflect distinct molecular mechanisms underlying the two myocardial infarction subtypes, warranting further investigation to elucidate the biological significance of this variation.

#### 3.1.3 *NBN* Expression

**Figure 2:**
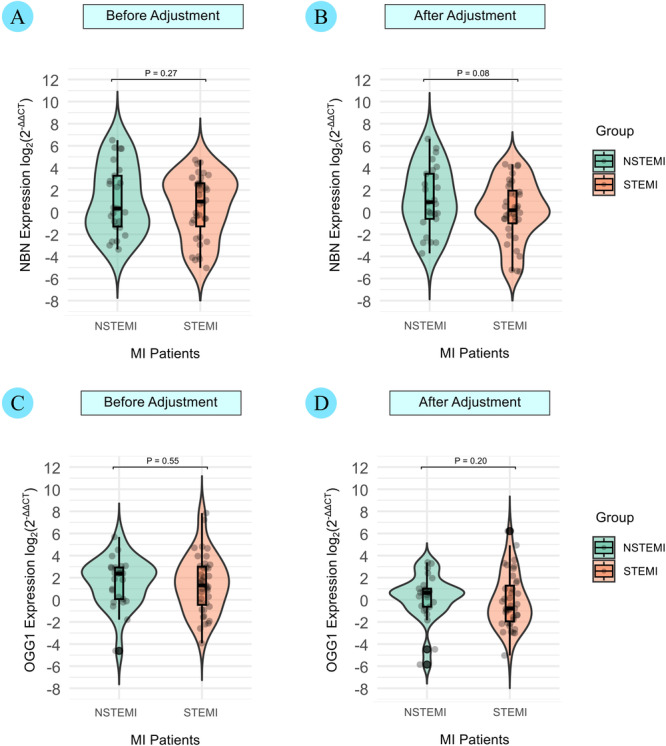
NBN and OGG1 gene expression in STEMI and NSTEMI patients.(A) and (B): The box plots represent relative expression of NBN gene (log2 (2^−ΔΔCt^)) in STEMI and NSTEMI patients before (A) and after (B) adjustment for potential confounders. (C) and (D): The box plots represent relative expression of OGG1 gene (log2 (2^−ΔΔCt^)) in STEMI and NSTEMI patients before (C) and after (D) adjustment for potential confounders (*, **, and *** represent *p* < 0.05, < 0.01, and < 0.001 respectively).

Before adjustment, the median log‐transformed NBN expression was higher in STEMI patients (1.14) compared to NSTEMI patients (0.34) (Figure 2A). However, this difference was not statistically significant (*p* = 0.27). After adjusting for potential confounders (Figure 2B), the median NBN expression slightly increased in NSTEMI group. Notably, the *p*‐value decreased to 0.08, still statistically not significant.

#### 3.1.4 *OGG1* Expression

Before adjustment (Figure 2C), the median log‐transformed OGG1 expression was higher in NSTEMI patients (2.627) compared with STEMI patients (1.30), although this difference was not statistically significant (*p* = 0.27). After adjusting for potential confounders (Figure 2D), the median OGG1 expression increased slightly in both groups, and the *p*‐value decreased to 0.08, approaching but not reaching statistical significance.

### 3.2 Co‐Expression Analysis of DNA Damage Repair Genes in STEMI and NSTEMI Patients

**Figure 3:**
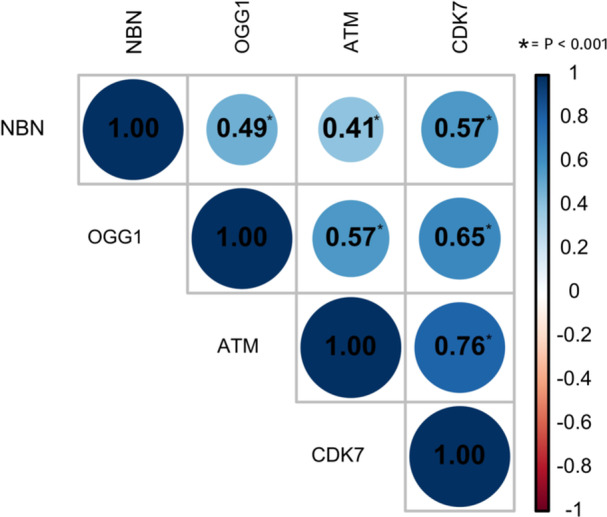
Pairwise Spearman correlation matrix of DDR gene expression (NBN, OGG1, ATM, and CDK7) in the study cohort. The size and color intensity of the circles represent the strength of correlation (blue = positive correlation, red = negative correlation) (* indicate statistically significant correlations at *p* < 0.001).

The co‐expression analysis of four DNA damage repair–related genes, NBN, OGG1, ATM, and CDK7 in whole‐blood samples from NSTEMI and STEMI patients revealed consistently positive and statistically significant correlations (*p* < 0.001 for all pairs) (Figure 3). NBN exhibited moderate positive correlations with OGG1 (r = 0.49), ATM (r = 0.41), and CDK7 (r = 0.57). OGG1 demonstrated moderate to strong correlations with ATM (r = 0.57) and CDK7 (r = 0.65). The strongest association was observed between ATM and CDK7 (r = 0.76). These findings indicate coordinated regulation of these genes in acute myocardial infarction, with ATM and CDK7 showing particularly high co‐expression, potentially reflecting shared regulatory mechanisms or functional interactions in DNA damage repair pathways.

### 3.3 Diagnostic Performance of CDK7

To assess whether CDK7 expression could clinically distinguish STEMI from NSTEMI, ROC analysis was performed. The area under the curve (AUC) was 0.63 (95% CI: 0.52–0.77), (Figure S01) indicating poor discriminatory ability. Adding age and sex to a logistic regression model improved the AUC to 0.70 (95% CI: 0.58–0.82), but this improvement was not statistically significant (ΔAUC = +0.07; *p* = 0.18). Therefore, CDK7 alone is not a robust clinical discriminator, and our findings should be interpreted as exploratory.

## 4 Discussion

Myocardial infarction (MI) is a major contributor to cardiovascular mortality worldwide, with its two major clinical subtypes STEMI and NSTEMI, differing in severity, pathophysiology, and prognosis. While substantial advances have been made in understanding the molecular mechanisms of MI in Western populations, such investigations remain scarce in South Asian populations, particularly in Bangladesh. In this study, we focused on comparing the expression of four key DNA damage response (DDR) genes, ATM, CDK7, OGG1, and NBN, between STEMI and NSTEMI patients to identify possible molecular differences between these subtypes in our population. MI induces oxidative stress and DNA damage, activating DDR pathways, which trigger inflammatory responses and influence cardiomyocyte survival, myocardial remodeling, and repair [11]. Although activation of these pathways is protective in the acute phase, excessive or prolonged DDR activation may exacerbate injury by amplifying inflammation [12]. Because DDR activity can be shaped by genetic background, DDR genes hold potential for developing population‐specific diagnostic and therapeutic strategies. In our study, ATM, OGG1, and NBN expression did not differ significantly between STEMI and NSTEMI patients, either before or after adjustment for confounders. The absence of a detectable difference in our cohort may reflect population‐specific factors, including genetic variation, environmental influences, or differences in ischemia duration and severity. It is also possible that these genes are equally activated in both subtypes, as both STEMI and NSTEMI trigger substantial oxidative stress and DNA damage. By contrast, CDK7 expression showed a clear difference: it was significantly higher in NSTEMI patients compared to STEMI patients, both before and after adjustment. CDK7 is a cyclin‐dependent kinase that functions as part of the transcription factor TFIIH complex, regulating transcription initiation and cell cycle progression. Its inhibition has been shown to improve cardiac remodeling and outcomes in experimental heart failure models [13]. Though, it was previously [5] reported that CDK7 expression was lower in NSTEMI patients compared to healthy controls, suggesting that while NSTEMI patients may have reduced CDK7 relative to normal baseline levels, they still exhibit higher expression than STEMI patients. This pattern may be explained by the differing ischemic profiles of STEMI and NSTEMI. STEMI is characterized by complete coronary artery occlusion, leading to more extensive myocardial necrosis and potentially stronger transcriptional repression. NSTEMI, typically caused by partial occlusion or distal embolization, results in prolonged but less complete ischemia, which may permit a more sustained transcriptional response. Higher CDK7 expression in NSTEMI patients could therefore reflect an adaptive attempt to maintain transcriptional and repair processes under moderate but persistent ischemic stress, whereas the severe ischemia of STEMI may overwhelm these mechanisms, leading to downregulation. However, CDK7 showed only modest ability to distinguish STEMI from NSTEMI (AUC = 0.63), indicating limited standalone diagnostic performance. Although adjustment for age and sex slightly improved discrimination (AUC = 0.70), this increase was not statistically significant, supporting that CDK7 is not a reliable independent clinical classifier and should be interpreted in an exploratory context. Nevertheless, the observed differential expression of CDK7 between STEMI and NSTEMI in the Bangladeshi population may reflect subtype‐specific transcriptional responses to ischemic injury and warrants further investigation in larger cohorts. The co‐expression analysis further supports the interconnected nature of these DDR genes in acute MI. In whole‐blood samples from STEMI and NSTEMI patients, all gene pairs showed positive and statistically significant correlations. NBN exhibited moderate correlations with OGG1, ATM, and CDK7. OGG1 showed moderate to strong correlations with ATM and CDK7. The strongest correlation was between ATM and CDK7, suggesting particularly close regulatory or functional interactions. The high co‐expression of ATM–CDK7 is noteworthy, given their distinct yet complementary roles in DNA damage sensing and transcriptional regulation, and it may indicate coordinated responses to ischemia‐induced DNA damage. In this study, peripheral blood was used as the source material for gene expression analysis in STEMI and NSTEMI patients because it is minimally invasive, readily accessible, and capable of reflecting systemic molecular changes following myocardial infarction. Previous studies have shown that whole blood (PBMCs) reliably capture post‐MI gene expression patterns [14, 15]. These systemic signatures can differ in magnitude between STEMI, which involves complete coronary occlusion, and NSTEMI, typically caused by partial occlusion. Moreover, circulating RNA in peripheral blood enables the identification of disease‐specific gene expression profiles, and such profiles have been shown to distinguish MI patients from healthy individuals while providing insight into disease progression [16]. This evidence supports the suitability of peripheral blood as a proxy for detecting and comparing DDR‐related gene expression differences between STEMI and NSTEMI.

To our knowledge, this is the first study in the Bangladeshi population to investigate the role of DDR pathways in distinguishing ST‐elevation subtypes of myocardial infarction. The novelty of this work lies in examining gene expression profiles of Bangladeshi MI patients to differentiate between STEMI and NSTEMI, an approach not previously reported for this population. By focusing on DDR gene expression, the study provides new molecular insights into subtype‐specific differences and highlights the potential for identifying novel biomarkers and therapeutic targets, thereby contributing to the development of precision cardiology in genomic research.

Despite these insights, it is important to acknowledge several limitations of this study. The small sample size may have limited the statistical power to detect subtle differences, particularly for ATM, OGG1, and NBN. In addition, as RNA was extracted from whole blood without cell‐type deconvolution, observed expression patterns may be influenced by fluctuations in underlying leukocyte proportions rather than purely intracellular regulation; so, these findings need to be looked at more closely in specific cell types. Furthermore, it is important to consider the potential impact of pre‐analytical and clinical heterogeneity on the results. While all blood samples in this study were collected within a standardized window of 4 hour post‐admission, the time elapsed from the actual onset of symptoms to hospital arrival may vary systematically between AMI subtypes. NSTEMI often presents with a less abrupt coronary occlusion and subtler clinical symptoms compared to the complete occlusion seen in STEMI, which can lead to later hospital presentation and a different transcriptional snapshot at the time of sampling. Additionally, acute pharmacological interventions, such as heparin or antiplatelet therapy administered prior to sampling, could potentially modulate the expression of DDR genes in peripheral blood cells. As specific data on symptom‐to‐sampling intervals and pre‐sampling medication were not available, their influence cannot be entirely ruled out. Future longitudinal studies with precise mapping of symptom onset and treatment timing are necessary to separate these clinical variables from the primary biological drivers of DDR gene expression. Moreover, the observational and cross‐sectional study design prevents us from drawing conclusions about causal relationships between gene expression changes and MI subtypes. Future research should incorporate functional assays and validation in larger cohorts, ideally including myocardial tissue samples, to confirm these associations. This will be crucial in determining whether CDK7 modulation has therapeutic relevance in myocardial infarction.

To better contextualize our findings within the broader landscape of acute coronary syndrome (ACS) diagnostics, it is important to compare our blood‐based transcriptomic approach with contemporary biomarker and multi‐marker strategies. Recent large‐scale clinical studies have focused on protein‐based cardiac and systemic biomarkers for cardiovascular risk stratification and NSTEMI rule‐out, demonstrating high diagnostic accuracy, particularly for hs‐cTnT and cMyBP‐C (AUC ~ 0.92) [17]. Multi‐marker approaches combining hs‐cTnT with cMyBP‐C or copeptin have further improved diagnostic and prognostic performance compared with exploratory upstream molecular markers [18, 19]. In contrast, CDK7 in our study showed only modest discriminatory ability between STEMI and NSTEMI, supporting the idea that peripheral blood expression of upstream cellular stress‐response genes may function more as mechanistic indicators than as high‐acuity diagnostic biomarkers. Furthermore, incorporation of biomarker panels into clinical algorithms such as the GRACE 1.0 score has also improved long‐term risk prediction in NSTE‐ACS patients [20]. Several factors may explain why ATM, OGG1, and NBN showed no significant subtype‐specific differences, while CDK7 demonstrated subtle divergence. Previous studies primarily evaluated stable circulating proteins released from necrotic myocardium, whereas our study measured intracellular leukocyte mRNA expression. In addition, protein biomarkers reflect direct myocardial injury, while DDR transcripts represent upstream and transient cellular stress responses that may be influenced by systemic inflammation. Differences in study population, timing of sample collection, and clinical endpoints may also contribute, as prior studies examined large Western cohorts with serial early sampling, whereas our study evaluated a Bangladeshi cohort at a single 4‐h post‐admission time point. Collectively, these findings suggest that whole‐blood transcriptomic signatures such as CDK7 may provide mechanistic and population‐specific insights into ACS pathophysiology, although they are not immediate substitutes for established necrosis‐based protein biomarkers.

## 5 Conclusion

In summary, our findings reveal distinct expression patterns of DNA damage response genes in STEMI and NSTEMI patients where CDK7 showed consistently higher levels in NSTEMI compared to STEMI. This suggests that CDK7 may reflect subtype‐specific transcriptional activity and warrants further investigation as a potential exploratory molecular marker in acute myocardial infarction. The observed gene co‐expression further supports the interconnected role of DDR pathways in post‐MI processes. Together, these results emphasize the potential of blood‐based gene expression profiling for improving our understanding of MI subtypes, although validation in larger cohorts and functional studies will be necessary before clinical translation.

## Ethics Statement

Ethical approval for this study was obtained from the Ethical Approval Committee of the Faculty of Biological Sciences, University of Dhaka.

## Consent

All participants filled out a questionnaire to gather their demographic information and health measurements by consent.

## Conflicts of Interest

The authors declare no conflicts of interest.

## Supporting information

Supporting File 1

Supporting File 2

## Data Availability

The data that support the findings of this study are available from the corresponding author upon reasonable request.
