# Supplementary figures and images for: CDK7 as a Potential Exploratory Biomarker for Distinguishing Acute Myocardial Infarction Subtypes via DDR Pathways: Evidence From a Bangladeshi Cohort
Source: Clin Cardiol. 2026 Jun 10;49(6):e70383. doi: 10.1002/clc.70383 (PMC13250832; doi:10.1002/clc.70383)

## Slide 1
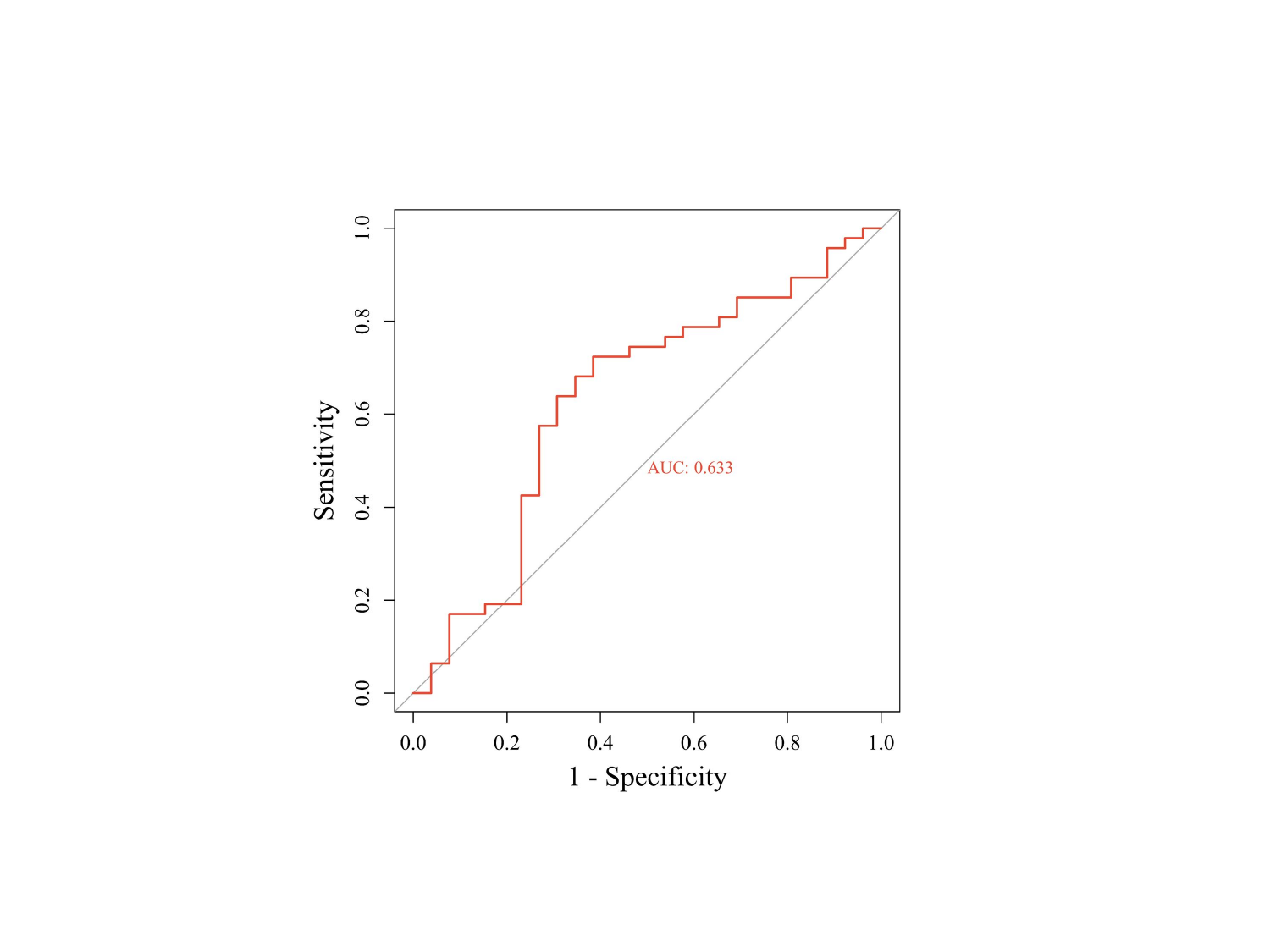

Supplement: Supplementary file 1 — Supporting File 1 [file CLC-49-e70383-s001.pptx]
