# Supplementary material for: CDK7 as a Potential Exploratory Biomarker for Distinguishing Acute Myocardial Infarction Subtypes via DDR Pathways: Evidence From a Bangladeshi Cohort
Source: Clin Cardiol. 2026 Jun 10;49(6):e70383. doi: 10.1002/clc.70383 (PMC13250832; doi:10.1002/clc.70383)
Supplement: Supplementary file 2 — Supporting File 2 [file CLC-49-e70383-s002.docx]

**Supplementary Table 1:** Primers predicted by the NCBI primer blast tool to amplify ATM, NBN, OGG1, CDK7 and GAPDH.

| **Gene** | **Forward Primer (5´->3´)** | **Reverse primer (5´->3´)** | **Annealing Temperature** | **Amplicon size(bp)** | **Target region(Accession)** |
| --- | --- | --- | --- | --- | --- |
| ATM | CAAACGAAATCTCAGTGATATTGACC | AGTGCCTTCTTCCACTCCTTTCAG | 60℃ | 100 | NM_001351834.2 |
| NBN | AATGGATATGCTCCAAAGGCAAGGT | TGGGGCGCTTGGCATTAGTTT | 62℃ | 193 | NM_002485.5 |
| OGG1 | GTTCCTCCAACAACAACATCGC | TGAGATGAGCCTCCACCTCTG | 60℃ | 153 | NM_002542.6 |
| CDK7 | AGGATGTATGGTGTAGGTGTGGA | AAGATGTGATGCAAAGGTATTCC | 60℃ | 221 | NM_001799.4 |
| GAPDH | TCAGCCGCATCTTCTTTTGC | CCCAATACGACCAAATCCGT | 60℃ | 88 | NM_002046.7 |
